# Feeding patterns and nutrition status of children aged 0–24 months in Rwanda: a secondary data analysis of Rwanda DHS 2019–2020

**DOI:** 10.1038/s41598-026-46969-x

**Published:** 2026-04-03

**Authors:** Edith Uwamahoro, Michael Habtu, Gashaija Absolomon, Jeanine Condo

**Affiliations:** 1 School of Public Health, University of Rwanda, Kigali, Rwanda; 2 Center for Impact, Innovation and Capacity Building in Health Information Systems and Nutrition (CIIC-HIN), Kigali, Rwanda; 3 Tulane School of Public Health and Tropical Medicine, New Orleans, LA USA

**Keywords:** Feeding patterns, Nutritional status, Child undernutrition, Children aged below 24 months, Demographic health surveys, Rwanda, Diseases, Health care, Medical research, Risk factors

## Abstract

Undernutrition among children under five remains a major global public health concern. In Rwanda, 33% of children under five are stunted despite extensive nutrition programs. Limited evidence exists examining how infant and young child feeding (IYCF) patterns relate to nutritional outcomes among children aged 0–24 months using population-based data. This study assessed feeding patterns and their association with nutritional status among children aged 0–24 months in Rwanda. Secondary analysis of the 2019–2020 Rwanda Demographic and Health Survey included 1,581 children aged 0–24 months with complete anthropometric and IYCF indicators data. IYCF practices were assessed using WHO/UNICEF indicators. Nutritional status indicators (stunting, wasting, underweight) were calculated using WHO 2006 Child Growth Standards. Multivariable logistic regression identified independent predictors of child malnutrition. Among 1,581 children, the prevalence of stunting was 28%, underweight 6.6%, and wasting 1.6%. Breastfeeding practices were strong (early initiation 85.5% and exclusive breastfeeding 81.4%), but complementary feeding remained inadequate (34.2% met minimum dietary diversity, 46% minimum meal frequency, and 22.4% minimum acceptable diet). Independent predictors included rural residence, low household wealth, single maternal status, male sex, and low birth weight for stunting; male sex, low birth weight, and recent diarrhea for underweight; recent fever and paradoxically meeting the minimum acceptable diet for wasting. This study’s findings underscore the need for integrated interventions improving complementary feeding, healthcare access, childhood illness prevention, and targeted support for high-risk children to reduce stunting, underweight, and wasting in Rwanda.

## Introduction

Undernutrition among children under five years remains a critical public health concern globally, accounting for approximately 45% of child deaths^1^. Despite global progress, stunting and wasting continue to disproportionately affect low- and middle-income countries (LMICs), while also being home to about 6.6 billion people, roughly four in five of the world’s population, bear nearly 89% of the global burden^2^. Sub-Saharan Africa (SSA) accounts for one-third of the world’s stunted children, with prevalence in the East African region, including Rwanda, exceeding 37%^3–6^. Optimal infant and young child feeding (IYCF) practices such as early initiation of breastfeeding within one hour of birth, exclusive breastfeeding for the first six months, timely introduction of complementary feeding, and continued breastfeeding up to two years are essential to achieve healthy growth and development^7,8^. The World Health Organization and UNICEF emphasize these practices as central to meeting global nutrition targets by 2025^9^. Inadequate dietary diversity and meal frequency increase children’s vulnerability to undernutrition, illness, and developmental deficits^5,6,10,11^.

Suboptimal IYCF practices remain prevalent across LMICs. Globally, only 29% of children consume food from the minimum number of food groups, with poorer households and rural communities particularly disadvantaged^5,6,12,13^. Poor feeding practices, coupled with poverty and limited access to health services, have been linked to stunting, wasting, and micronutrient deficiencies^7^. Studies in Tanzania and Nigeria have shown that low minimum dietary diversity (MDD), minimum meal frequency (MMF), and minimum acceptable diet (MAD) significantly increase the odds of child undernutrition^14–17^.

In Rwanda, despite extensive nutrition programs, undernutrition remains high. According to the 2019–2020 Demographic and Health Survey, 33% of children under five were stunted, 8% underweight, and 1% wasted^18^. While breastfeeding indicators are encouraging, complementary feeding practices remain inadequate in Rwanda, and existing studies have not sufficiently examined how infant and young child feeding patterns, including breastfeeding practices, dietary diversity, and feeding frequency relate to nutritional outcomes among children aged below 24 months using population-based data.

Understanding how these determinants interact is crucial to inform evidence-based interventions. This study was therefore designed to determine the factors associated with childhood nutritional status by assessing feeding patterns among children aged below 24 months in Rwanda. Specifically, the study sought to: (1) determine the nutritional status of children aged below 24 months in Rwanda; (2) assess their feeding patterns; and (3) examine the association between feeding patterns and nutritional status, focusing on wasting, underweight, and stunting. This study makes several important contributions to the evidence base: (1) it provides population-level data on the specific IYCF-nutrition linkages among children aged 0–24 months in Rwanda, a critical period for growth and development; (2) it examines multiple nutritional outcomes (stunting, wasting, and underweight) simultaneously, allowing for comprehensive understanding of feeding practice impacts; and (3) it addresses the gap in evidence on how specific IYCF indicators (MDD, MMF, MAD) relate to nutritional status in the Rwandan context, where breastfeeding practices are strong but complementary feeding remains suboptimal. These findings are expected to generate critical evidence to guide the design and implementation of policies and programs that address persistent undernutrition, promote optimal feeding practices, and support healthy child growth and development in Rwanda.

## Methods

### Study design and data source

This study used secondary data from the 2019–2020 Rwanda Demographic and Health Survey (RDHS, 2019–2020) to assess the association between infant and young child feeding practices and nutritional status among children aged 0–24 months. The RDHS is a nationally representative, cross-sectional household survey conducted every five years by the National Institute of Statistics of Rwanda (NISR) and the Ministry of Health, with technical support from ICF International (Rockville, MD, USA) under the Demographic and Health Surveys (DHS) Program. RDHS 2019–2020 fieldwork was conducted between November 2019 and July 2020.

A two-stage stratified sampling design was applied using the 2012 Rwanda Population and Housing Census as the sampling frame^19^. In the first stage, 500 enumeration areas (villages) were selected with probability proportional to population size and stratified by district. In the second stage, 26 households were systematically selected from each cluster, yielding approximately 13,000 households. Sampling weights provided in the DHS datasets were applied to ensure representativeness at national and sub-national levels. Response rates were 99.7% for women and 99.5% for men.

For this analysis, two DHS datasets were merged: the Kids Recode (KR) file, containing data on children’s health, feeding, and anthropometry, and the Household Recode (PR) file, containing household-level characteristics. After merging, the analytic sample comprised 1,581 children aged 0–24 months with complete anthropometric and IYCF indicators data.

### Study population and eligibility criteria

Children aged 0–24 months with valid anthropometric measurements and complete IYCF indicators information were included. Children outside this age range or with missing key data were excluded from the analysis.

### Study variables

The study outcome variables included child nutritional status indicators, stunting (height-for-age z score < − 2 SD), wasting (weight-for-height z score < − 2 SD), underweight (weight-for-age z score < − 2 SD), and overweight (weight-for-height z score > + 2 SD) were derived according to WHO 2006 Child Growth Standards using WHO Anthro software (version 3.2.2, January 2011) and the WHO Anthro macro for SPSS (World Health Organization, Geneva, Switzerland). Each indicator was coded dichotomously (1 = Yes: affected; 0 = No: normal). Low birth weight (LBW) was defined as < 2,500 g. Child anemia was defined as hemoglobin < 11.0 g/dL (altitude-adjusted per DHS/WHO conventions).

The study’s explanatory variables were IYCF practices, assessed using WHO/UNICEF-recommended indicators, including early initiation of breastfeeding within one hour of birth and exclusive breastfeeding for the first six months of life, timely introduction of complementary foods, continued breastfeeding up to 2 years, and the WHO-defined composite indicators: Minimum Dietary Diversity (MDD), Minimum Meal Frequency (MMF), and Minimum Acceptable Diet (MAD). MDD/MMF/MAD were constructed using the DHS 24-hour dietary recall questions following WHO/UNICEF indicator guidance^20^.

### Potential confounders

Child, maternal, and household characteristics considered potential confounders included: child age, sex, birth weight, recent diarrhea (within 2 weeks before the survey), anemia status, maternal age, education, occupation, body-mass index (BMI), antenatal-care visits, place of delivery, exposure to breastfeeding counseling, postnatal check-ups, vitamin A and micronutrient-powder supplementation, household wealth index, type of residence (urban/rural), region, and religion. Vitamin A supplementation was defined as receipt of a vitamin A dose in the last 6 months for age-eligible children (vitamin A programs begin at approximately 6 months of age). Multiple micronutrient powder (MNP; locally known as “Ongera”) was captured as receipt/consumption within the DHS recall period for age-eligible children. Rotavirus vaccination was categorized as having received all 3 doses versus not, based on vaccination information from the child health card and/or caregiver report. Preceding birth interval applied only to multiparous mothers; primiparous mothers were excluded from birth interval categorization.

### Statistical analysis

The data were analyzed using Stata version 17.1 (Stata Corp, College Station, TX, USA). Descriptive statistics summarized participant characteristics and feeding practices. All analyses accounted for sampling weights, clustering, and stratification to ensure nationally representative estimates. Bivariate logistic regression was conducted to assess the relationship between each explanatory variable and the nutritional outcomes. Variables with *p* ≤ 0.05 in the bivariate analysis were included in the multivariable logistic regression model to identify independent predictors while adjusting for confounders. Reference categories for categorical variables in the regression models were selected based on the category expected to have the lowest risk of malnutrition or representing the most favorable condition (e.g., rich household wealth, female sex, normal birth weight, urban residence). Adjusted odds ratios (AOR) with 95% confidence intervals (CI) were calculated. Model diagnostics included tests for multicollinearity, independence of observations, and linearity of continuous variables. Statistical significance was set at *p* < 0.05. All results are presented with appropriate measures of variability (standard deviation [SD] or standard error [SE]).

### Ethical consideration

This secondary analysis utilized publicly available, de-identified DHS data obtained with permission from the DHS Program (Rockville, MD, USA). Ethical approval for this analysis was also obtained from the College of Medicine and Health Sciences Institutional Review Board, University of Rwanda (Ref: CMHS/IRB/525/2024).

## Results

### Study population characteristics

Table 1 summarizes the sociodemographic characteristics of 1,581 children aged 0–24 months, their mothers, and households included in the study. Nearly half of the children were aged 12–23 months (49.6%). Most resided in rural areas (80.3%), and the gender distribution was nearly equal (52.0% male and 48.0% female). Most children had normal birth weight (≥ 2500 g) (94.4%). Recent childhood morbidities included diarrhea (19.9%) and fever (23.9%), while anemia was prevalent (52.8%). Coverage of preventive interventions varied, with 63.6% of children having received vitamin A supplementation in the six months preceding the survey and only 14.2% having received multiple micronutrient powders in the previous seven days. Most children (92.1%) had received three doses of rotavirus vaccination by the time of the survey, whereas deworming coverage in the preceding six months was moderate (47.2%). Postnatal care attendance within two months after delivery was low (18.7%).

Most mothers were aged 20–34 years (67.0%), had less than secondary education (72.7%), were employed (78.6%), and married or living with a partner (82.8%). Most had normal BMI (68.8%). Maternal health services utilization showed high levels of health facility delivery (94.4%), about half of mothers attended four or more antenatal care visits (46.6%), and nearly one-third of mothers did not receive breastfeeding counseling (30.6%). Preceding birth intervals were predominantly 60 months (38.6%), with shorter intervals being less common. Household wealth distribution showed a higher proportion of households in the poor category (44.2%) compared with middle (18.9%) and rich (36.8%) categories.

**Table 1:** Sociodemographic and maternal characteristics of children aged 0–24 months, Rwanda DHS 2019–2020 (*n* = 1174).

Variable	*N* (%)
Child age group	
> 6 months	377 (23.9)
6–8 months	206 (13.1)
9–11 months	212 (13.4)
12–23 months	783 (49.6)
Gender	
Male	821 (52.0)
Female	759 (48.0)
Child birthweight	
Low birthweight	89 (5.6)
Normal birthweight	1491 (94.4)
Had diarrhea 2 weeks prior to the survey	315 (19.9)
The child got vitamin A in the last 6 months	1005 (63.6)
The child got Multiple Micronutrient Powder (Ongera)	225 (14.2)
The child received 3 doses of the rotavirus vaccine	1400 (92.1)
Had fever	378 (23.9)
The child received deworming drugs	746 (47.2)
Child’s anemia status	
Anemic	633 (52.8)
Not anemic	567 (47.3)
Post-natal check of the baby within 2 months	287 (18.7)
Religion	
Christian	1521 (96.3)
Muslim	35 (2.2)
Other	24 (1.5)
Region	
Kigali	182 (11.5)
South	380 (24.1)
West	401 (25.4)
North	245 (15.5)
East	372 (23.5)
Residence	
Urban	312 (19.7)
Rural	1268 (80.3)
Wealth index	(0.0)
Poor	699 (44.2)
Middle	299 (18.9)
Rich	582 (36.8)
Maternal age	
< 20 Years	50 (3.2)
20–34 Years	1058 (67.0)
> 34	472 (29.9)
Maternal education	
Less than secondary	1148 (72.7)
Secondary or higher	432 (27.3)
Maternal occupation	
Not working/farmer	338 (21.4)
Employed	1242 (78.6)
Marital status	
Married or living with partner	1309 (82.8)
Single	163 (10.3)
Widowed	19 (1.2)
Divorced or separated	89 (5.6)
Maternal BMI	
Underweight	58 (3.7)
Normal Weight	1086 (68.8)
Overweight	324 (20.5)
Obesity	110 (7.0)
Antenatal visit (ANC)	
No ANC	41 (2.7)
One ANC Visit	68 (4.4)
Two or Three ANC Visits	710 (46.3)
Four ANC visits and above	715 (46.6)
Place of delivery	
Home	66 (4.2)
Health facility	1492 (94.4)
Other	22 (1.4)
Breastfeeding counseling by a healthcare provider	
Didn’t get breastfeeding counseling	483 (30.6)
Got breastfeeding counseling	1097 (69.4)
Women empowerment	
Earn more than him	80 (11.4)
Earn less than him	428 (60.8)
Earn almost the same	183 (26.0)
Partner does not bring in money	11 (1.6)
Do not know	2 (0.3)
Proceeding birth interval	
7–23 months	74 (6.3)
24-35 months	143 (12.2)
36-47 months	216 (18.4)
48-59 months	288 (24.5)
60 + months	453 (38.6)

Frequency (n); Percentage (%); LBW cutoff (≥ 2500 g), anemia cutoffs (Hb < 110 g/L for 6–59 months; <105 g/L for < 6 months), preceding birth interval calculated among multiparous women only (N = 1,174); primiparous women (n = 406, 25.7%) excluded; time periods for vitamin A (6 months), MNP (7 days), rotavirus vaccine (by survey), and deworming (6 months). The DHS wealth index is originally constructed as five quintiles using principal component analysis of household assets; for this analysis, quintiles were collapsed into three categories: ‘Poor’ (lowest two quintiles combined), ‘Middle’ (middle quintile), and ‘Rich’ (upper two quintiles combined).

### Nutritional status and feeding patterns

Table 2 presents the nutritional status and feeding practices of children aged 0–24 months. Stunting was observed in 28.0% of children, underweight in 6.6%, wasting in 1.6%, and overweight in 1.0%. Nearly all children had been breastfed (99.7%), with early initiation within one hour of birth practiced by 85.5%. Among infants aged 0–5 months, exclusive breastfeeding was reported for 81.4%. Timely introduction of complementary foods at 6–8 months occurred in 80.1%, and continued breastfeeding at 12–23 months was practiced by 86.6%. Among children aged 6–23 months, only 34.2% met the minimum dietary diversity, 46.0% met the minimum meal frequency, and 22.4% met the minimum acceptable diet.

**Table 2:** Child health characteristics and nutritional status of children aged 0–24 months in Rwanda, DHS 2019–2020 (*n* = 1,581).

Variable	*N* (%)
Childhood stunting status	440 (28)
Childhood wasting status	25 (1.6)
Childhood underweight status	104 (6.6)
Childhood overweight status	16 (1)
Introduced to solid, semi-solid, or soft foods at 6 months	165 (80.1)
Exclusive breastfeeding within the first 6 months	307 (81.4)
Ever breastfed	1529 (99.7)
Initiation of breastfeeding in the 1 h of birth	1311 (85.5)
Minimum dietary diversity (MDD)	412 (34.2)
Minimum meal frequency (MMF)	553 (46)
Minimum acceptable diet (MAD)	269 (22.4)
Continued breastfeeding to 2 years	678 (86.6)

Frequency (n); Percentage (%).

### Bivariate associations with child malnutrition

Table 3 presents the bivariate associations between child, maternal, and household factors and nutritional outcomes. Wasting was more common among male children (*p* < 0.001), children with low birth weight (*p* = 0.021), children who had recent fever (*p* = 0.018), and, unexpectedly, children meeting the minimum acceptable diet (*p* = 0.024). Regional variation in wasting was observed, with the highest prevalence in Kigali and the South (*p* = 0.040).

Underweight was more frequent among children from rural areas (*p* = 0.001), poorer households (*p* = 0.004), children of mothers with lower education (*p* = 0.001) or low body mass index (*p* = 0.008), male children (*p* < 0.001), children with low birth weight (*p* < 0.001), and children who had recent fever (*p* = 0.002) or diarrhea (*p* < 0.001). Exclusive breastfeeding (*p* = 0.005) and meeting minimum meal frequency (*p* = 0.009) were associated with lower underweight prevalence.

Stunting was more prevalent among children from rural areas (*p* < 0.001), poor households (*p* < 0.001), children of mothers with lower education (*p* < 0.001) or low BMI (*p* < 0.001), male children (*p* < 0.001), older children aged 12–23 months (*p* < 0.001), children with low birth weight (*p* < 0.001), and children who had recent diarrhea (*p* = 0.007). Children of older mothers (> 34 years) had higher stunting prevalence compared to adolescent mothers (*p* = 0.041), and regional disparities were observed, with the highest prevalence in the North region (*p* = 0.001). Protective factors against stunting included attending four or more antenatal care visits (*p* = 0.001), receiving breastfeeding counseling (*p* = 0.037), rotavirus vaccination (*p* = 0.014), deworming (*p* < 0.001), vitamin A supplementation (*p* < 0.001), consumption of multi-micronutrient powders (*p* < 0.001), and meeting minimum dietary diversity (*p* = 0.021), minimum meal frequency (*p* = 0.026), or minimum acceptable diet (*p* = 0.005).

**Table 3:** Bivariate analysis: determinants of childhood nutrition status among children aged 0–24 months in Rwanda (RDHS 2019–2020).

Variable	Child wasting		*P*-value	Child underweight		*P*-value	Child stunting		*P*-value
	No, *n* (%)	Yes, *n* (%)		No, *n* (%)	Yes, *n* (%)		No, *n* (%)	Yes, *n* (%)	
Religion			0.491			0.405			0.760
Christian	1489 (98)	23 (2)		1417 (94)	98 (6)		1089 (72)	425 (28)	
Muslim	34 (97)	1 (3)		32 (91)	3 (9)		26 (74)	9 (26)	
Other	23 (96)	1 (4)		20 (87)	3 (13)		17 (74)	6 (26)	
Region			**0.040**			0.720			**0.001**
Kigali	174 (97)	5 (3)		172 (95)	9 (5)		142 (79)	38 (21)	
South	366 (97)	11 (3)		349 (92)	29 (8)		272 (72)	106 (28)	
West	396 (99)	3 (1)		376 (94)	23 (6)		277 (69)	123 (31)	
North	244 (100)	1 (0)		228 (93)	17 (7)		157 (64)	88 (36)	
East	366 (99)	5 (1)		344 (93)	26 (7)		284 (77)	85 (23)	
Residence			0.287			**0.001**			**< 0.001**
Urban	301 (98)	7 (2)		302 (97)	8 (3)		258 (84)	50 (16)	
Rural	1245 (99)	18 (1)		1167 (92)	96 (8)		874 (69)	390 (31)	
Wealth index			0.772			**0.004**			**< 0.001**
Poor	686 (98)	11 (2)		636 (91)	62 (9)		444 (64)	253 (36)	
Middle	290 (98)	6 (2)		278 (94)	17 (6)		211 (71)	85 (29)	
Rich	570 (99)	8 (1)		555 (96)	25 (4)		477 (82)	102 (18)	
Maternal age			0.760			0.127			**0.041**
< 20 years	49 (98)	1 (2)		48 (96)	2 (4)		41 (82)	9 (18)	
20–34 years	1036 (99)	15 (1)		990 (94)	62 (6)		770 (73)	282 (27)	
> 34 years	461 (98)	9 (2)		431 (92)	40 (8)		321 (68)	149 (32)	
Maternal education			0.713			**0.001**			**< 0.001**
< Secondary	1124 (98)	19 (2)		1054 (92)	90 (8)		790 (69)	354 (31)	
≥ Secondary	422 (99)	6 (1)		415 (97)	14 (3)		342 (80)	86 (20)	
Marital status			0.509			0.816			**0.080**
Married/living with partner	1282 (99)	19 (1)		1218 (93)	85 (7)		954 (73)	348 (27)	
Single	159 (98)	3 (2)		152 (94)	10 (6)		110 (68)	52 (32)	
Widowed	19 (100)	0 (0)		18 (95)	1 (5)		12 (63)	7 (37)	
Divorced/separated	86 (97)	3 (3)		81 (91)	8 (9)		56 (63)	33 (37)	
Maternal BMI			0.163			**0.008**			**< 0.001**
Underweight	55 (95)	3 (5)		51 (88)	7 (12)		34 (59)	24 (41)	
Normal Weight	1066 (99)	16 (1)		1001 (92)	82 (8)		756 (70)	329 (30)	
Overweight	318 (99)	4 (1)		309 (96)	13 (4)		252 (79)	68 (21)	
Obesity	107 (98)	2 (2)		108 (98)	2 (2)		90 (83)	19 (17)	
Antenatal visits			0.472			0.058			**0.001**
No ANC	40 (98)	1 (2)		37 (90)	4 (10)		27 (66)	14 (34)	
1 Visit	67 (99)	1 (1)		60 (88)	8 (12)		42 (62)	26 (38)	
2–3 Visits	698 (99)	7 (1)		658 (93)	48 (7)		491 (70)	215 (30)	
4 + Visits	697 (98)	14 (2)		678 (95)	34 (5)		548 (77)	163 (23)	
Breastfeeding counselling (yes/no)			0.469			0.849			**0.037**
Yes	1071 (98)	19 (2)		1018 (93)	73 (7)		802 (74)	288 (26)	
Rotavirus vaccination			0.095			0.485			**0.014**
Not vaccinated	112 (97)	4 (3)		111 (95)	6 (5)		96 (81)	22 (19)	
Vaccinated	1375 (99)	20 (1)		1301 (93)	95 (7)		987 (71)	408 (29)	
Deworming (yes/no)			0.051			0.144			**< 0.001**
Yes	736 (99)	7 (1)		702 (94)	42 (6)		491 (66)	253 (34)	
Child age group			0.312			0.326			**< 0.001**
< 6 Months	366 (98)	7 (2)		354 (95)	20 (5)		309 (83)	65 (17)	
6–8 Months	198 (97)	6 (3)		186 (91)	19 (9)		157 (77)	48 (23)	
9–11 Months	207 (99)	3 (1)		195 (93)	15 (7)		169 (80)	41 (20)	
12–23 Months	773 (99)	9 (1)		732 (94)	50 (6)		496 (64)	285 (36)	
Sex of child			**< 0.001**			**< 0.001**			**< 0.001**
Male	804 (98)	13 (2)		745 (91)	73 (9)		537 (66)	280 (34)	
Female	742 (98)	12 (2)		724 (96)	31 (4)		595 (79)	160 (21)	
Birth weight			**0.021**			**< 0.001**			**< 0.001**
Low birthweight	83 (95)	4 (5)		65 (75)	22 (25)		41 (47)	47 (53)	
Normal	1463 (99)	21 (1)		1404 (94)	82 (6)		1091 (74)	393 (26)	
Fever (past 2 weeks)			**0.018**			**0.002**			0.157
Yes	365 (97)	11 (3)		339 (90)	38 (10)		260 (69)	116 (31)	
Diarrhea (past 2 weeks)			0.621			**< 0.001**			**0.007**
Yes	309 (99)	4 (1)		276 (88)	38 (12)		207 (66)	107 (34)	
Vitamin A supplementation			0.101			0.792			**< 0.001**
Yes	988 (99)	12 (1)		937 (94)	65 (6)		686 (68)	316 (32)	
Multi-micronutrient powder			0.363			0.365			**< 0.001**
Yes	223 (99)	2 (1)		207 (92)	18 (8)		137 (61)	88 (39)	
Exclusive breastfeeding			0.719			**0.005**			**< 0.001**
Yes	421 (99)	6 (1)		413 (96)	16 (4)		340 (79)	88 (21)	
Minimum dietary diversity (MDD)			0.155			0.253			**0.021**
Yes	401 (98)	9 (2)		387 (94)	24 (6)		300 (73)	111 (27)	
Minimum meal frequency (MMF)			0.408			**0.009**			**0.026**
Yes	540 (98)	10 (2)		522 (95)	27 (5)		395 (72)	154 (28)	
Minimum acceptable diet (MAD)			**0.024**			0.117			**0.005**
Yes	260 (97)	8 (3)		255 (95)	13 (5)		203 (76)	65 (24)	

Weighted chi-square tests accounting for complex survey design. Some significant p-values reflect large sample size rather than large effect sizes. Unadjusted associations; confounding controlled in Table 4. Regional disparities: chi-square across 5 provinces. Bold p-values indicate statistical significance at *p* < 0.05.

### Independent predictors of child malnutrition

Multivariable logistic regression for stunting identified six independent predictors: rural residence (AOR = 1.72, 95% CI: 1.12–2.64, *p* = 0.013), middle wealth quintile (AOR = 0.61, 95% CI: 0.42–0.88, *p* = 0.007), rich wealth quintile (AOR = 0.49, 95% CI: 0.33–0.72, *p* < 0.001), single motherhood (AOR = 1.63, 95% CI: 1.03–2.57, *p* = 0.036), female sex (AOR = 0.49, 95% CI: 0.37–0.65, *p* < 0.001), and normal birth weight (AOR = 0.51, 95% CI: 0.29–0.91, *p* = 0.023). Borderline significant associations were observed for maternal age > 34 years (AOR = 2.48, 95% CI: 0.96–6.36, *p* = 0.060), breastfeeding counselling (AOR = 0.76, 95% CI: 0.56–1.04, *p* = 0.089), and East region (AOR = 0.57, 95% CI: 0.32–1.02, *p* = 0.057).

Multivariable logistic regression for underweight identified three independent predictors: female sex (AOR = 0.39, 95% CI: 0.23–0.65, *p* < 0.001), normal birth weight (AOR = 0.24, 95% CI: 0.12–0.47, *p* < 0.001), and recent diarrhea in the two weeks preceding the survey (AOR = 1.86, 95% CI: 1.12–3.08, *p* = 0.016). Middle wealth quintile showed a borderline protective association (AOR = 0.54, 95% CI: 0.28–1.06, *p* = 0.075).

Multivariable logistic regression for wasting identified two independent predictors: recent fever in the two weeks preceding the survey (AOR = 3.69, 95% CI: 1.42–9.56, *p* = 0.007) and paradoxically, meeting minimum acceptable diet criteria (AOR = 3.11, 95% CI: 1.20–8.10, *p* = 0.020), Several factors that showed significant bivariate associations lost statistical significance after adjustment in multivariable models, including vitamin A supplementation, multi-micronutrient powder consumption, exclusive breastfeeding, antenatal care visits, deworming, and individual IYCF indicators (MDD, MMF) for stunting and underweight outcomes as shown in Table 4.

**Table 4:** Multivariable logistic regression analysis of independent predictors of childhood malnutrition among children aged 0–24 months in Rwanda, DHS 2019–2020 (*n* = 1,581).

Variable	AOR	95% CI	*P*-value
Child stunting			
Region			
Kigali	Ref	–	–
South	0.63	0.35–1.12	0.115
West	0.92	0.52–1.62	0.774
North	1.13	0.62–2.05	0.698
East	0.57	0.32–1.02	0.057
Residence			
Urban	Ref	–	–
Rural	1.72	1.12–2.64	**0.013**
Wealth index			
Poor	Ref	–	–
Middle	0.61	0.42–0.88	**0.007**
Rich	0.49	0.33–0.72	**< 0.001**
Maternal age			
< 20 years	Ref	–	–
20–34 years	1.79	0.72–4.43	0.210
> 34 years	2.48	0.96–6.36	0.060
Maternal education			
Less than secondary	Ref	–	–
Secondary or higher	0.98	0.68–1.43	0.931
Marital status			
Married/living with partner	Ref	–	–
Single	1.63	1.03–2.57	**0.036**
Widowed	1.58	0.50–4.99	0.436
Divorced/separated	1.60	0.92–2.80	0.099
Maternal BMI			
Underweight	Ref	–	–
Normal weight	0.92	0.50–1.70	0.785
Overweight	0.97	0.48–1.94	0.924
Obesity	0.88	0.36–2.13	0.772
Antenatal visits			
No Antenatal Visit	Ref	–	–
1 Visit	1.80	0.66–4.89	0.248
2–3 Visits	0.95	0.41–2.17	0.903
4 + Visits	0.74	0.32–1.71	0.478
Breastfeeding counseling			
No	Ref	–	–
Yes	0.76	0.56–1.04	0.089
Deworming			
No	Ref	–	–
Yes	0.98	0.68–1.40	0.908
Sex of child			
Male	Ref	–	–
Female	0.49	0.37–0.65	**< 0.001**
Birth weight			
Low birth weight	Ref	–	–
Normal birth weight	0.51	0.29–0.91	**0.023**
Diarrhea (past 2 weeks)			
No	Ref	–	–
Yes	1.19	0.87–1.62	0.285
Vitamin A supplementation			
No	Ref	–	–
Yes	0.97	0.65–1.46	0.894
Multi-micronutrient powder			
No	Ref	–	–
Yes	1.31	0.93–1.85	0.125
Exclusive breastfeeding			
No	Ref	–	–
Yes	0.96	0.62–1.49	0.857
Minimum dietary diversity (MDD)			
No	Ref	–	–
Yes	0.99	0.65–1.51	0.948
Minimum meal frequency (MMF)			
No	Ref	–	–
Yes	1.03	0.73–1.45	0.874
Minimum acceptable diet (MAD)			
No	Ref	–	–
Yes	0.86	0.49–1.52	0.601
Child underweight			
Residence			
Urban	Ref	–	–
Rural	1.78	0.78–4.03	0.169
Wealth index			
Poor	Ref	–	–
Middle	0.54	0.28–1.06	0.075
Rich	0.89	0.47–1.69	0.729
Maternal education			
Less than secondary	Ref	–	–
Secondary or higher	0.68	0.35–1.34	0.271
Maternal BMI			
Underweight	Ref	–	–
Normal weight	0.71	0.28–1.83	0.480
Overweight	0.57	0.19–1.75	0.328
Obesity	0.30	0.05–1.76	0.183
Fever (past 2 weeks)			
No	Ref	–	–
Yes	1.25	0.75–2.07	0.385
Sex of child			
Male	Ref	–	–
Female	0.39	0.23–0.65	**< 0.001**
Birth weight			
Low birth weight	Ref	–	–
Normal birth weight	0.24	0.12–0.47	**< 0.001**
Diarrhea (past 2 weeks)			
No	Ref	–	–
Yes	1.86	1.12–3.08	**0.016**
Exclusive breastfeeding			
No	Ref	–	–
Yes	0.87	0.41–1.85	0.721
Minimum meal frequency (MMF)			
No	Ref	–	–
Yes	0.71	0.43–1.16	0.173
Child wasting			
Religion			
Christian	Ref	–	–
Muslim	2.00	0.25–16.31	0.517
Other	4.62	0.55–38.69	0.159
Fever (past 2 weeks)			
No	Ref	–	–
Yes	3.69	1.42–9.56	**0.007**
Sex of child			
Male	Ref	–	–
Female	0.89	0.34–2.29	0.805
Birth weight			
Low birth weight	Ref	–	–
Normal birth weight	0.43	0.09–1.98	0.280
Minimum acceptable diet (MAD)			
No	Ref	–	–
Yes	3.11	1.2–8.1	**0.020**

Reference categories (baseline for comparison): rich wealth, female sex, normal birth weight (≥ 2,500 g), urban, married/partnered. AOR = Adjusted Odds Ratio; CI = Confidence Interval. ORs > 1.0 = risk factors; <1.0 = protective. Model fit: Hosmer-Lem show χ^2^=8.45, *p* = 0.39; Pseudo R^2^ = 0.328. Bold p-values indicate statistical significance at *p* < 0.05.

## Discussion

This secondary analysis of the 2019–2020 Rwanda Demographic and Health Survey provides national evidence on factors influencing the nutritional status of children aged 0–24 months. The findings indicate that child undernutrition in Rwanda remains a multifactorial problem shaped by socioeconomic, maternal, and child characteristics, as well as feeding practices and recent illness. Children living in rural areas were significantly more likely to be stunted compared with their urban counterparts, consistent with previous studies in sub-Saharan Africa showing that rural residence is associated with limited access to diverse diets, poor sanitation, and restricted healthcare access^1,7^. Stunting was also inversely related to household wealth and maternal education, emphasizing the well-established influence of socioeconomic conditions on child growth^21,22^. Wealthier households are better able to afford nutritious foods, maintain hygiene, and access healthcare, contributing to improved child nutrition outcomes. The finding that children of single mothers were more likely to have stunted children aligns with evidence that single-parent households often face greater economic insecurity and reduced social support, limiting adequate feeding and care^23^.

The strong association between recent fever and child wasting underscores the role of infection in precipitating acute malnutrition. Fever, often indicative of malaria or other infections, can lead to nutrient depletion, appetite loss, and impaired absorption, resulting in rapid weight loss^24–27^. Similar associations have been observed across sub-Saharan Africa and South Asia, reinforcing the importance of integrating nutrition interventions with infection control and child health services^24,28^.

An unexpected finding in this study was the higher likelihood of wasting among children meeting the Minimum Acceptable Diet (MAD). Supplementary analysis examining the relationship between wasting and consumption of animal source foods (a key component of diet quality) revealed that among wasted children, 28.3% consumed animal source foods compared to 31.2% of non-wasted children (*p* = 0.68), suggesting that animal protein intake alone did not explain this paradox. Additionally, examination of the timing of MAD achievement relative to wasting status suggests that some children may have had their diets improved following clinical recognition of wasting, representing reverse causation rather than MAD causing wasting. Among the 25 wasted children in our sample, the proportion meeting MAD was higher than expected (32% vs. 22.4% overall), which may reflect caregivers’ response to visible weight loss by improving dietary diversity and frequency. This aligns with recent critiques in the literature regarding the limitations of the MAD indicator. While MAD ensures basic dietary diversity and feeding frequency, it does not fully capture the nutritional adequacy needed to prevent wasting, particularly in terms of protein quality, essential fatty acids, and micronutrient intake^11,29,30^. A study done in Bangladesh found that even children who adhered to MAD were vulnerable to wasting due to the limited availability of nutrient-dense foods, underscoring the need for more comprehensive dietary indicators beyond MAD alone^31^. These results suggest the need to revisit and strengthen dietary indicators used for programmatic monitoring to better reflect nutrient adequacy rather than only frequency and diversity.

The context of civil conflict and displacement in Rwanda’s history continues to have indirect effects on child nutrition outcomes. While the current analysis focuses on 2019–2020 data more than two decades after the 1994 genocide against the Tutsi, regional disparities in undernutrition may partly reflect long-term impacts of past displacement, disrupted livelihoods, and ongoing internal migration patterns. Areas that experienced significant displacement historically may continue to show elevated malnutrition rates due to intergenerational effects on household wealth accumulation, land ownership, and access to resources. Additionally, periodic population movements related to economic opportunities and resettlement programs can affect household food security and access to health services. Future research examining temporal trends in specific regions could help elucidate how these historical and ongoing displacement patterns influence current nutrition outcomes.

The present study findings also reaffirm the significance of maternal and household factors in shaping child nutrition outcomes. Maternal education remains a consistent predictor of positive nutrition practices and healthcare utilization^32,33^, while maternal undernutrition has been associated with low birthweight and subsequent child growth failure^21,34,35^.

Gender disparities in malnutrition among children, where boys are more susceptible to growth disturbances and frequent infections, continue to be observed. Other studies reported similar gender differences, attributing the increased risk in males to both biological factors and caregiving practices that may favor girls in some settings^36,37^. Additionally, low weight at birth was recognized to be associated with underweight. Further analysis found that children born with low weight significantly experience more growth failure and underweight status linked to early nutritional deficits and compromised immune development^24,38–40^.

### Study limitations

This study has several limitations. First, the cross-sectional design precludes causal inference and cannot establish temporal relationships, particularly for the paradoxical MAD-wasting association which likely reflects reverse causality. Second, while multivariable logistic regression was used, more advanced statistical approaches such as path analysis or structural equation modeling (SEM) could better elucidate complex pathways, mediating effects, and interactions among determinants; future studies should employ these methods. Third, 24-hour dietary recall is subject to recall bias and may not represent usual intake patterns. Fourth, unmeasured confounders (household food security, water/sanitation quality, maternal mental health, intra-household food distribution) may influence observed associations. Fifth, the low wasting prevalence (1.6%, *n* = 25) limited statistical power, producing wide confidence intervals. Finally, the MAD indicator’s inability to capture nutrient density, portion sizes, and food quality constrains dietary adequacy assessment.

### Study strengths

This study has notable strengths. First, it utilized nationally representative data from the 2019–2020 Rwanda DHS with standardized methodology, ensuring generalizability. Second, the large sample size (*n* = 1,581) provided adequate statistical power for detecting associations. Third, comprehensive assessment of multiple domains (socioeconomic, maternal, child, feeding, and health factors) enabled robust multivariable analysis. Fourth, use of WHO-standardized anthropometric and IYCF indicators ensures international comparability. Fifth, the study addresses a critical knowledge gap by examining the 0-24-month window, the critical period for preventing irreversible growth faltering. Finally, rigorous statistical approaches with appropriate model diagnostics strengthen confidence in findings.

### Implications for policy and practice

First, targeted interventions must prioritize high-risk populations: rural communities, poor households, single-mother families, and male children. Second, complementary feeding programs should emphasize nutrient quality beyond diversity and frequency, with specific focus on increasing access to animal source foods and energy-dense foods. Third, integration of nutrition and health services is essential, particularly linking complementary feeding support with infection prevention and treatment programs given the strong fever-wasting and diarrhea-underweight associations. Fourth, community health worker programs should be strengthened to provide counseling on both quantity and quality of complementary foods, with particular attention to feeding during illness. Fifth, improving women’s nutrition, education, and empowerment remains essential to break the intergenerational cycle of malnutrition. Finally, continued investment in rural health infrastructure and nutrition-sensitive agriculture are critical to improving dietary quality and child growth outcomes.

Future research should employ longitudinal designs to establish causal pathways, particularly for the MAD-wasting relationship. Development of enhanced dietary quality indicators beyond current IYCF metrics is needed to better capture nutrient adequacy and animal source food consumption. Studies should further examine how historical displacement patterns influence current nutrition outcomes in specific regions.

## Conclusion

Stunting remains a significant public health concern among children aged 0–24 months in Rwanda, affecting 28% of children, while underweight (6.6%) and wasting (1.6%) are less prevalent. While breastfeeding practices are strong (exclusive breastfeeding 81.4%, early initiation 85.5%), critical gaps exist in complementary feeding quality, with only 34.2% meeting minimum dietary diversity, 46% meeting minimum meal frequency, and 22.4% meeting minimum acceptable diet criteria. Key risk factors independently associated with undernutrition include rural residence, household poverty, single maternal status, male sex, low birthweight, and recent illness. The paradoxical association between meeting minimum acceptable diet criteria and wasting highlights potential limitations of current IYCF indicators in capturing nutritional adequacy, particularly regarding protein quality and nutrient density. Findings suggest that integrated interventions may benefit from enhancing the quality and nutrient density of complementary foods with particular emphasis on increasing access to animal source foods, integrating nutrition services with infection prevention and treatment programs, and targeting high-risk populations including rural communities, poor households, and single-mother families. Strengthening monitoring systems to better assess dietary quality beyond current indicators will support evidence-based programming to reduce child malnutrition in Rwanda.

## Data Availability

The datasets analyzed during the current study are publicly available from the DHS Program repository (https://dhsprogram.com/data/available-datasets.cfm) upon registration and reasonable request. Researchers interested in accessing the data must submit a request through the DHS Program website and agree to the data use terms and conditions. The specific dataset used is Rwanda DHS 2019-2020 Final Report (FR370).

## Abbreviations

ANC: Antenatal care
AOR: Adjusted odds ratio
BMI: Body mass index
CI: Confidence interval
CMHS: College of medicine and health sciences
DHS: Demographic and health survey
ICF: ICF international
IYCF: Infant and young child feeding
LBW: Low birth weight
LMICs: Low- and middle-income countries
MAD: Minimum acceptable diet
MDD: Minimum dietary diversity
MMF: Minimum meal frequency
NISR: National Institute of Statistics of Rwanda
RDHS: Rwanda demographic and health survey
SD: Standard deviation
SSA: Sub-Saharan Africa
UNICEF: United Nations International Children’s Fund
WASH: Water, sanitation, and hygiene
WHO: World Health Organization
